# Data collection of patients with diabetes in family medicine: a study in north-eastern Italy

**DOI:** 10.1186/s12913-017-2508-5

**Published:** 2017-08-16

**Authors:** Alberto Vaona, Franco Del Zotti, Sandro Girotto, Claudio Marafetti, Giulio Rigon, Alessandro Marcon

**Affiliations:** 1 Federazione Italiana Medici di Medicina Generale (FIMMG), Centro Studi FIMMG Verona, Verona, Italy; 2 Unit of Epidemiology and Medical Statistics, Department of Diagnostics and Public Health, University of Verona, c/o Istituti Biologici II, Strada Le Grazie 8, 37134 Verona, Italy

**Keywords:** Diabetes, Family medicine, Data collection, Q-score, Performance, Primary care

## Abstract

**Background:**

Studies on data collection and quality of care in Italian family medicine are lacking. The aim of this study was to assess the completeness of data collection of patients with diabetes in a large sample of family physicians in the province of Verona, Veneto region, a benchmark for the Italian National Health System.

**Methods:**

We extracted the data on all the patients with diabetes from the electronic health records of 270 family physicians in 2006 and 2009. We reported the percentage of patients with data recorded for 12 indicators of performance derived from the National Institute for Clinical Excellence diabetes guidelines. Secondarily, we assessed quality of care using the Q-score (the lower the score, the greater the risk of cardiovascular events).

**Results:**

Patients with diabetes were 18,507 in 2006 and 20,744 in 2009, and the percentage of patients registered as having diabetes was 4.9% and 5.4% of the total population, respectively (*p* < 0.001). Data collection improved for all the indicators between 2006 and 2009 but the performance was still low at the end of the study period: patients with no data recorded were 42% in 2006 and 32% in 2009, while patients with data recorded for ≥5 indicators were 9% in 2006 and 17% in 2009. The Q-score improved (mean ± SD, 20.7 ± 3.0 in 2006 vs 21.3 ± 3.6 in 2009, *p* < 0.001) but most patients were at increased risk of cardiovascular events in both years (Q-score ≤ 20).

**Conclusions:**

We documented an improvement in data collection and quality of care for patients with diabetes during the study period. Nonetheless, data collection was still unsatisfactory in comparison with international benchmarks in 2009. Structural interventions in the organization of family medicine, which have not been implemented since the study period, should be prioritised in Italy.

**Electronic supplementary material:**

The online version of this article (doi:10.1186/s12913-017-2508-5) contains supplementary material, which is available to authorized users.

## Background

Beyond its clinical and social burden, diabetes also has a dramatic impact on the consumption of economic resources [1, 2]. Several studies showed that evidence-based management of diabetes and good quality of patient care can contribute to slow disease progression, reduce the economic costs and achieve better patients’ outcomes [3–5].

A complete and proper collection of patients’ data is the basis for an effective set up of clinical improvement strategies [6]. Table 1 reports data on the clinical management of diabetes derived from national or local surveys in Italy, UK and Australia during the years 2003–2009 [7–11]. The United Kingdom (UK) diabetes national audit program has become an international benchmark, since it has covered the whole country for a long period and it has been carried out regularly [7]. All currently available data about diabetes management in Italian family medicine were collected through surveys on primary care physicians involved on a voluntary basis. For this reason, findings from these studies may depend on self-selection of particularly careful physicians, motivated to improve their performance.

**Table 1:** Non-systematic review of evidence on data collection performance in primary care of diabetes derived from national and international benchmark cross sectional studies

Country, year [reference]	Italy, 2004 [8]	Italy, 2008 [9]	Italy, 2006 [10]	Italy, 2009 [10]	UK, 2005–6 [7]	UK, 2006–7 [7]	UK, 2007–8 [7]	Australia, 2008 [11]
Area extension	Local (Ascoli)	Local (Ravenna)	Country wide	Country wide	Country wide	Country wide	Country wide	Country wide
Voluntary recruitment	Yes	Yes	Yes	Yes	Yes	Yes	Yes	Yes
N physicians involved	26	6	755	862	-	-	5359	20
Reference period (months)	12	12	12	12	12	12	12	15
Diabetes prevalence (%)	5.3	5.4	7.4	6.7	-	3.8	3.9	6.5
Blood pressure (%)^a^	-	24	44.7	44.5	86	91	94	88
BMI (%)^a^	-	13	32.2	38.7	81	85	88	-
Smoking habits (%)^a^	-	-	74.3	83.6	79	86	87	-
Total Cholesterol (%)^a^	-	-	-	-	-	-	-	-
LDL Cholesterol (%)^a^	70	40	24.1	33.7	81	88	90	87
Microalbuminuria (%)^a^	-	26	22.4	30.2	48	60	63	-
Creatinine (%)^a^	-	-	66.1	66.7	83	89	91	-
Creatinine clearance (%)^a^	-	-	-	-	-	-	-	-
Glycated haemoglobin (%)^a^	50	39	60.2	63.3	83	88	91	-
Foot examination (%)^a^	2	-	-	-	64	72	78	-
Eye examination (%)^a^	25	-	42.9	42.0	61	66	67	-
Urine test (%)	-	-	-	-	-	-	-	-

*BMI* body mass index, *LDL* low-density lipoprotein

^a^Percentage of patients with diabetes having ≥1 registration in the last year

In Italy a mixed public-private system provides healthcare. The public part, the National Health System, is administrated on a regional basis and responsibility for healthcare delivery is given by each Region to public companies (local healthcare trust, LHT). Italian citizens receive healthcare services from the LHTs, and primary and secondary diabetes healthcare are ensured for free.

In the province of Verona, in Northern Italy (Additional file 1: Figure S1), one LHT (Azienda Ulss 9 Scaligera) provides services to about 900,000 inhabitants http://statistica.regione.veneto.it/banche_dati_societa_residenti_eta_sesso.jsp. Several studies have documented that the prevalence of diabetes in the area of Verona is about 5.5–5.9% [5, 12, 13]. In this area, on initiative of the major family physicians’ Union (*FIMMG Verona*), a central databank was implemented during 2008 in order to remotely collect clinical performance data from professional databases of members of the Union. These data were used to address Union’s policy towards the economic agreements with the LHTs. Between 2006 and 2009 about 650 family physicians were active in the province of Verona; 270 (42%) of them were FIMMG members and they were all connected to the databank. These family physicians received benefits coming from economy of scale in their purchases of professional equipment and, mostly, from remote real-time assistance on auditing their clinical or prescribing performance targets, which were linked to economic incentives. The central databank was actively collecting data until 2010, when it was finally switched off. During the 2006–2009 time period no economic agreement on family physicians’ clinical performance on diabetes was active.

The primary aim of this study is to assess data collection for diabetes in family medicine in the province of Verona in 2006 and 2009 and to compare it with available data from national and international studies. The secondary aim is to explore the quality of care using the Q-score, a score predicting the development of cardiovascular events.

## Methods

### Study design

All 270 family physicians working in the province of Verona, who adhered to the databank in 2008, were included in this study. The data on all their patients with diabetes, which had been recorded by the family physicians during every day real-life practice in their professional electronic databases, were collected for 2006 and 2009 after irreversible anonymization.

Patients with diabetes were identified considering either a reported classification of diabetes according to the International Classification of Diseases 9th edition, or an exemption from health spending from diabetes, or “diabetes” as free text. An analysis of data consistency and data entry quality had been performed when each family physician joined the databank, but there was no standardisation of diabetes definition.

### Data collection performance indicators

Indicators of performance were chosen *ex-post* from the National Institute for Clinical Excellence (NICE) 2005 diabetes guidelines, which are also the reference in the UK diabetes national audit program [7, 14, 15]. These guidelines were selected as follows. Among 21 diabetes clinical guidelines available in 2006, 3 guidelines reported performance indicators (see Additional file 1: Table S1). Three independent reviewers rated these guidelines according to a score obtained by a three-item checklist [16]. The guidelines with the highest mean score were selected.

Among the indicators available in the NICE guidelines, we pragmatically selected the 12 indicators with data available in our study: blood pressure, body mass index (BMI), smoking habits, total cholesterol, low-density lipoprotein (LDL) cholesterol, microalbuminuria (MA), creatinine, creatinine clearance, glycated haemoglobin, foot examination, eye examination and urine test. For each indicator, we collected data on the number of registrations per year in the family physicians’ electronic health records. According to the guidelines, at least 1 registration per year had to be recorded for each indicator to be at target performance, except for blood pressure and glycated haemoglobin, where at least 4 registrations per year were required. Data on the measured values were available only for the last examination registered.

### Quality of diabetes care score

In order to assess the quality of diabetes care, we computed the Q-score, which predicts the development of cardiovascular events. The Q-score was developed and validated in a sample of the Italian population [3]. It ranges between 0 and 40 (lowest to highest quality of care). It has been reported that a Q-score ≤ 10 predicts an 89% greater risk of long-term cardiovascular events as compared to a score > 20 [3]. Briefly, a 0 to 10 score was assigned to every patient with available information on each of 4 dimensions: glycated haemoglobin, blood pressure, low-density lipoprotein (LDL) and microalbuminuria (MA). For each dimension, the assigned score reflected both the values of the indicators measured at the last examination registered and the presence/absence of at least one examination in the index year. Patients with incomplete information or non-valid data on one or more of the above indicators were excluded from this assessment.

### Statistical analysis

Categorical variables were described as percentages, and quantitative variables were summarised as mean ± SD or median with interquartile range (1st and 3rd quartiles). Temporal variations in patients’ and family physicians’ characteristics were tested by the Wilcoxon matched-pairs signed-ranks test and McNemar’s exact test as appropriate.

We appraised data collection from two points of view. In the first analysis, which illustrates the completeness of family physicians’ electronic databases, we reported the percentage of family physicians with 0%, ≥50% (between 50% and 100%) and ≥75% (between 75% and 100%) of their patients with data recorded for each indicator. In the second analysis, we considered family physicians as a single group, and we reported the percentage of patients with recorded data for each indicator, as well as the distribution of the patients according to the number of indicators recorded. This latter analysis focuses on patients, rather than on family physicians, and it shows the proportion of the overall population of patients with satisfactory data registration.

We tested temporal variations in performance using population-averaged, generalised estimating equations (GEEs) for a binomial outcome with logarithmic link, with family physicians identified as the clustering factor, and year (2009 versus 2006) as the main independent variable. These models provided crude risk ratios (RR). Adjusted RRs were obtained after additional adjustment for patients’ characteristics that could influence a family physician’s propensity to record data: sex, age (categorised as <40, 40–59, 60–79, ≥80 years to account for possible non-linear associations), antidiabetic therapy (1 = metformin or other hypoglycaemic agent (alone); 2 = insulin or analogue; 0 = none of them) as an indicator of disease severity, and additional therapy (1 = at least one among statin, acetylsalicylic acid or other platelet aggregation inhibitor, renin-angiotensin system modifier; 0 = none of them) as a proxy of comorbidities.

## Results

### Characteristics of the family physicians

Most of the 270 family physicians included in the study were men (*n* = 204, 76%) and they were aged 52 ± 5 years on average in 2006. The total number of patients in their databases was 373,827 and 385,962 in 2006 and 2009, respectively. The median number of patients per family physician was 1467 (1st and 3rd quartiles: 1225–1603) in 2006 and 1505 (1st and 3rd quartiles: 1289–1620) in 2009 (*p* < 0.001). The percentage of patients registered as having diabetes was 4.9% (95% CI: 4.7; 5.1) in 2006 and it increased to 5.4% (95% CI: 5.1; 5.6) in 2009 (*p* < 0.001).

### Characteristics of the diabetic patients

The number of patients with diabetes was 18,507 in 2006 and 20,744 in 2009, and the vast majority of them (*n* = 16,862) were cared for by the same family physician in both years. About half of the patients were men (53%) and their mean age was 66.1 and 67.8 years in 2006 and 2009 respectively (Table 2). In 2006, 26% of the patients were under oral antidiabetic therapy (mainly metformin). More than half were under lipid lowering, antiplatelet or antidiuretic drugs. For all the drugs considered, there was a substantial increase in the percentage of patients treated in 2009 vs 2006 (Table 2) (all *p* < 0.001).

**Table 2:** Demographic information and therapeutic regimen of patients with diabetes in 2006 and 2009^a^

		2006 (*N* = 18,507)	2009 (*N* = 20,744)	*p*-value
Demographic information	Male sex	9797 (53)	10,948 (53)	0.818
	Age (yr)	66.1 ± 13.7	67.8 ± 13.8	<0.001
Antidiabetic therapy	Metformin	2893 (16)	6989 (34)	<0.001
	Other hypoglycaemic agent	413 (2)	1348 (6)	<0.001
	Insulin (or analogue)	1994 (11)	3099 (15)	<0.001
	Any in the category	4771 (26)	9762 (47)	<0.001
Additional therapy	Statin	4040 (22)	7202 (35)	<0.001
	Acetylsalicylic acid or other platelet aggregation inhibitor	4951 (27)	7974 (38)	<0.001
	Renin-angiotensin system modifier	8255 (45)	11,815 (57)	<0.001
	Any in the category	10,097 (55)	14,610 (70)	<0.001

^a^Number (percentage) or mean ± SD reported

### Data collection performance of the family physicians

In 2006 the proportion of family physicians with no data recorded in their electronic databases ranged between 33%, for total cholesterol measurement, to 93%, for foot examination (Table 3). In 2009 the data collection improved for all the indicators and the family physicians with ≥50% or ≥75% of their patients with recorded data increased significantly for 5 and 3 of the 12 indicators, respectively.

**Table 3:** Percentage of the 270 general practitioners (family physicians) who had 0%, ≥50% or ≥75% of their patients with diabetes having data recorded for each of the 12 performance indicators in 2006 and 2009

Performance indicators	Percentage of the family physicians with 0% of their patients with data		Percentage of the family physicians with ≥50% of their patients with data		Percentage of the family physicians with ≥75% of their patients with data	
	2006	2009	2006	2009	2006	2009
Blood pressure^b^	49	38^***^	0	0	0	0
BMI^a^	35	19^***^	17	20	6	6
Smoking habits^a^	67	57^**^	1	1	1	1
Total cholesterol^a^	33	13^***^	24	44^***^	3	7^*^
LDL cholesterol^a^	47	35^***^	6	10^**^	0	0
Microalbuminuria^a^	56	37^***^	2	10^***^	0	0
Creatinine^a^	36	15^***^	24	42^***^	2	7^**^
Creatinine clearance^a^	76	47^***^	0	0	0	0
Glycated haemoglobin^b^	72	45^***^	0	0	0	0
Foot examination^a^	93	74^***^	0	0	0	0
Eye examination^a^	35	9^***^	0	0	0	0
Urine test^a^	38	17^***^	17	35^***^	2	5^*^

*BMI* body mass index, *LDL* low-density lipoprotein

^a^≥ 1 registration/year

^b^≥ 4 registrations/year

^*,**,***^ p for the comparison of proportions between years <0.05, <0.01, <0.001

A similar picture emerged when considering the family physicians as a single group (Table 4). In 2006, the proportion of patients with data on the indicators ranged between 0.2% (foot examination) and 30.6% (total cholesterol). Both the crude and adjusted analyses showed an increase in all the indicators, with the exception of blood pressure (≥4 registrations/year), which showed a significant 9% decrease (adjusted RR = 0.91; 95% CI: 0.85, 0.98) (Table 4). In the adjusted analysis, the relative change in performance for the other indicators ranged between a 17% increase for BMI registration (RR = 1.17; 95% CI: 1.14, 1.21) to a four-fold increase for foot examination (RR = 4.87; 95% CI: 2.97, 7.99).

**Table 4:** Number (percentage) of patients with diabetes having data recorded for each of the 12 indicators in 2006 and 2009, and relative increase (relative risk, RR, with 95% CI) in the proportion of patients with recorded data in 2009 with respect to 2006

	Performance indicator	2006 (*N* = 18,507)	2009 (*N* = 20,744)	2009 vs. 2006	2009 vs. 2006
		n (%)	n (%)	crude RR (95% CI)	adjusted^c^ RR (95% CI)
*a*	Blood pressure^b^	1267 (6.8%)	1470 (7.1%)	1.04 (0.97, 1.12)	0.91 (0.85, 0.98)
*b*	BMI^a^	4665 (25.2%)	6126 (29.5%)	1.27 (1.23, 1.32)	1.17 (1.14, 1.21)
*c*	Smoking habits^a^	730 (3.9%)	1034 (5.0%)	1.34 (1.21, 1.47)	1.25 (1.14, 1.37)
*d*	Total cholesterol^a^	5664 (30.6%)	8726 (42.1%)	1.37 (1.33, 1.41)	1.27 (1.23, 1.30)
*e*	LDL^a^	2427 (13.1%)	3829 (18.5%)	1.37 (1.30, 1.44)	1.21 (1.16, 1.27)
*f*	Microalbuminuria^a^	1093 (5.9%)	3438 (16.6%)	2.69 (2.41, 3.02)	2.33 (2.11, 2.57)
*g*	Creatinine^a^	5482 (29.6%)	8376 (40.4%)	1.36 (1.31, 1.40)	1.26 (1.22, 1.29)
*h*	Creatinine clearance^a^	173 (0.9%)	623 (3.0%)	2.90 (2.40, 3.51)	2.27 (1.90, 2.71)
*i*	Glycated haemoglobin^b^	200 (1.1%)	453 (2.2%)	2.03 (1.70, 2.42)	1.60 (1.34, 1.90)
*j*	Foot examination^a^	32 (0.2%)	194 (0.9%)	6.18 (3.70, 10.33)	4.87 (2.97, 7.99)
*k*	Eye examination^a^	2148 (11.6%)	3593 (17.3%)	1.53 (1.45, 1.62)	1.30 (1.24, 1.37)
*l*	Urine test^a^	4830 (26.1%)	7461 (36.0%)	1.36 (1.32, 1.41)	1.28 (1.24, 1.32)

*BMI* body mass index, *LDL* low-density lipoprotein

^a^≥ 1 registration/year

^b^≥ 4 registrations/year

^c^Adjusted for patient’s sex, age, antidiabetic therapy, other therapy

**Fig. 1:**
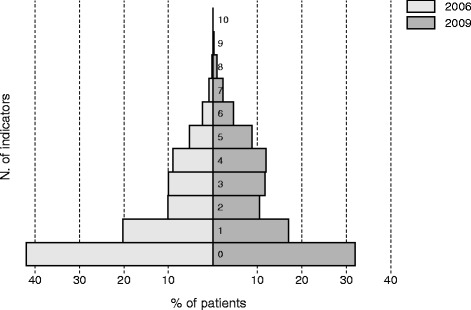
Distribution of the patients with diabetes according to the number of indicators recorded in 2006 and 2009

Despite these large relative improvements, the proportion of patients with data on the indicators in 2009 was still low from an absolute point of view (between 0.9%, for foot examination, and 42.1%, for total cholesterol). The percentage of patients with no indicators recorded was 42% in 2006 and it decreased to 32% in 2009 (Fig. 1). The percentage of patients with ≥5 indicators recorded was 9% in 2006 and 17% in 2009, but no diabetic patient had data for more than 10 indicators either in 2006 or 2009.

### Quality of diabetes care

Since we had no measured values for MA, patients who had performed the exam in the last year were assigned an intermediate score of 5, as done by the developers of the score [3], while the other patients were set to missing. An assessment of quality of care was possible for 14,480 (78%) patients in 2006 and 14,162 (68%) patients in 2009. The Q-score slightly increased in 2009 (mean ± SD, 21.3 ± 3.6) with respect to 2006 (20.7 ± 3.0) (*p* < 0.001). However, the large majority of patients had a score ≤ 20 (85% in 2006, vs. 76% in 2009) (Additional file 1: Table S2).

## Discussion

This study reports on data collection and quality of care for diabetes in family medicine in the area of Verona, located in the Veneto region of Italy, which is considered a benchmark region for quality and efficiency of care from the national Ministry of Health http://www.sanita24.ilsole24ore.com/pdf2010/Editrice/ILSOLE24ORE/QUOTIDIANO_SANITA/Online/_Oggetti_Correlati/Documenti/2017/02/02/NUOVO_BENCHMARK.pdf?uuid=AEaAZHM. https://www.regione.veneto.it/web/guest/comunicati-stampa/dettaglio-comunicati?_spp_detailId=3091057. http://www.sanita24.ilsole24ore.com/art/dal-governo/2017-02-02/marche-veneto-e-umbria-rosa-benchmark-181646.php?uuid=AEYX7tM. We found that the proportion of patients registered as having diabetes was 4.9% and 5.4% in 2006 and 2009 respectively, in agreement with the prevalence rates estimated in other studies in the same area [5, 12, 13].

Our analyses seem to show an improvement in data collection of patients with diabetes over the study period. We detected an increase in the proportion of family physicians with ≥50% and ≥75% of their patients with available data on the indicators; when considering the family physicians as a single group, we found a significant increase in data collection for all the performance indicators, with the exception of blood pressure recording (maybe due to the recommendation to opt for home measurement). All these improvements could be explained by a better recording style due to the data consistency and quality checks performed after the connection with the databank (occurred in 2008) but they could also be explained as the effect of educational programs or secular trends.

Despite these improvements, it is remarkable that, in absolute terms, the performance was still unsatisfactory in both years on all the indicators. A comparison with other data from Italy and from different countries (for the closest periods available) suggests that data collection in Italian family medicine was lagging behind international benchmarks (Table 1). In our study foot examination was the indicator with the worst performance: it was recorded just in 0.2% and 0.9% of the patients in 2006 and 2009 respectively. Performance for this indicator was 2% in the Ascoli benchmark for 2004 [8], while it was 72% and 78% in the UK in the 2006–7 and 2007–8 Diabetes National Audit, respectively [7]. We found that eye examination was carried out in 11.6% and 17.3% of the patients in 2006 and 2009 respectively, which is quite low compared to the Ascoli 2004 benchmark (25%) [8], to the Health Search national benchmark for 2006 and 2009 (43% and 42% respectively) [10], and especially to the UK benchmark for 2006–7 and 2007–8 (66% and 67% respectively) [7]. BMI measurement was recorded in 25.2% and 29.5% of our patients in 2006 and 2009, while the Italian Health Search and UK benchmarks were 32.2–37.8% and 85–88%, respectively [7, 10]. Blood pressure (2006: 6.8%, and 2009: 7.1%) and glycated haemoglobin measurements (2006: 1.1%, and 2009: 2.2%) also showed a poor performance. Four registrations/year were required to fulfil these demanding criteria and this probably contributed to the negative results. We found a scattered and sparse performance also for the number of indicators recorded, which we analysed as a marker of completeness of care (or “all-care process” indicator [7]): the registration decreased as the number of indicators considered increased.

This unsatisfactory level of performance and the gap compared to the international benchmarks are hard to explain with a lack of performance of individual family physicians, and we believe that health care organization is likely to play a relevant role [17]. To the authors’ knowledge, the vast majority of Italian family physicians work without supporting staff, medical assistants and nurses. The observed low global performance may be a consequence of poor teamwork organization and clinical work overload, because resources may be inadequate especially when the number of patients with identified type-2 diabetes increases, taking family physicians to a *plateau* of their capacity to follow up an increasing number of patients.

In the interpretation of our findings it is also necessary to consider that the effectiveness of electronic health record-based management of diabetes have been demonstrated primarily with process measures and selected intermediate outcomes, such as glycated haemoglobin and LDL levels, rather than clinical outcomes [18] but it is reasonable to believe that improvement of care strictly depends on the availability and quality of data [19]. In our study an available record for an indicator does not necessarily mean that its measured value is at target. For these reasons, it is relevant to highlight that the analysis of the validated Q-score confirms that the quality of diabetes clinical management is largely improvable. According to the Q-score, even if the proportion of patients with a low quality of care (Q-score ≤ 20) was slightly lower in 2009 than in 2006, most patients with diabetes were at increased risk of cardiovascular events and less than one patient out of four was receiving a good quality of care in 2009.

This study has several limitations. First, the data refer to several years ago. To our knowledge, however, this is the first study performed in Italy on an unselected sample of Italian family physicians. A central databank collected data from all the family physicians who were members of the FIMMG Union, which corresponded to approximately 42% of all the family physicians working in the study area. Since the Union membership is not reasonably associated with professional performance, our study design minimized the risk of self-selection bias with respect to previous studies on voluntary physicians. Thus, we believe that our study sample is representative of the real-life management of diabetes in the study area and period. Although the data are not recent, no major widespread changes in the organisation of family medicine have been implemented in Italy since the study period: the last National General Medical Service Contract was signed in 2005 http://www.sisac.info/anteprimaNewsHome.do?tipo=WEB&idArea=201011221610481056&idNews=201012212330479102&tit=&cat=&ddal=&dal=09/05/2017. http://www.sisac.info/elencoNewsArea.do?rproprietario=201012211450435842&rrubrica=0&rdataaccordo=&operazione=ricerca&dataDal=&idArea=201012211450435842&idPagina=1&colOrderBy=tnews_versioni.news_titolo&order=asc&ricercaTitolo=&numberOfRecordsPerPage=10. Sparse educational interventions might have improved data recording quality only modestly. Thus, the level of performance of family physicians is unlikely to have changed significantly since the study period. All the indicators selected were included in the following update of the NICE clinical guidelines [20].

A second limitation is that we could only report on data collection but we were not able to analyse the measured values of the selected indicators, because measurement data were patchy. Third, because of a lack of integration between primary and secondary health care levels, we are unable to distinguish between poor clinical management and missed registration of measurements or examinations. Doctors could have omitted to record some of their patients’ medical data that were already available in hospital databases (one third of the patients attended diabetes centres). Fourth, our disease definition was operational and pragmatic, formulated by family physicians for working purposes and it did not necessarily fit the guidelines definition. However, using data from another study on diabetes in Legnago (province of Verona) in 2009, we found that 1745 out of 2127 (82%) subjects defined as having diabetes by a similar disease definition were also identified on the basis of administrative data (disease exemptions, admission to diabetologic services, or purchase of antidiabetic drugs or glucose test strips) [5]. Also, we acknowledge that our disease definition could not disentangle between type-1 and type-2 diabetes, but patients with type-1 diabetes are a minority (5%) of the total population with diabetes and the same definition was used to extract patients for both years. Finally, the Q-score was calculated only for a part of the patients with available data.

## Conclusions

Data collection and quality of primary care for diabetes improved between 2006 and 2009 but they were still unsatisfactory in comparison with international benchmarks. Structural interventions in the organization of family medicine, which have not been implemented since the study period, should be prioritised in Italy in order to improve the management of patients with diabetes.

## Additional file

Additional file 1: Map of the study area (**Figure S1**); review of reference guidelines on diabetes management available in 2006 (**Table S1**); distribution of the Q-score in 2006 and 2009 (**Table S2**). (DOC 165 kb)

## Abbreviations

FIMMG: Federazione Italiana Medici di Medicina Generale
LDL: Low-density lipoprotein
LHT: Local healthcare trust
MA: Microalbuminuria
NICE: National Institute for Clinical Excellence
RR: Risk ratios
