# Supplementary material for: Data collection of patients with diabetes in family medicine: a study in north-eastern Italy
Source: BMC Health Serv Res. 2017 Aug 16;17:565. doi: 10.1186/s12913-017-2508-5 (PMC5559811; doi:10.1186/s12913-017-2508-5)
Supplement: Additional file 1: — Map of the study area (Figure S1); review of reference guidelines on diabetes management available in 2006 (Table S1); distribution of the Q-score in 2006 and 2009 (Table S2). (DOC 165 kb) [file 12913_2017_2508_MOESM1_ESM.doc]

**Additional file 1**

**Manuscript title:**

Data collection of patients with diabetes in family medicine: a study in north-eastern Italy

**Authors:**

Alberto Vaona, Franco Del Zotti, Sandro Girotto, Claudio Marafetti, Giulio Rigon, Alessandro Marcon

**Figure S1**: map of Italy. The study includes data from 42% (n=270) of all the GPs who were active in 2006-2009 in the province of Verona (hatched area), located in the Veneto region (white area).^*^


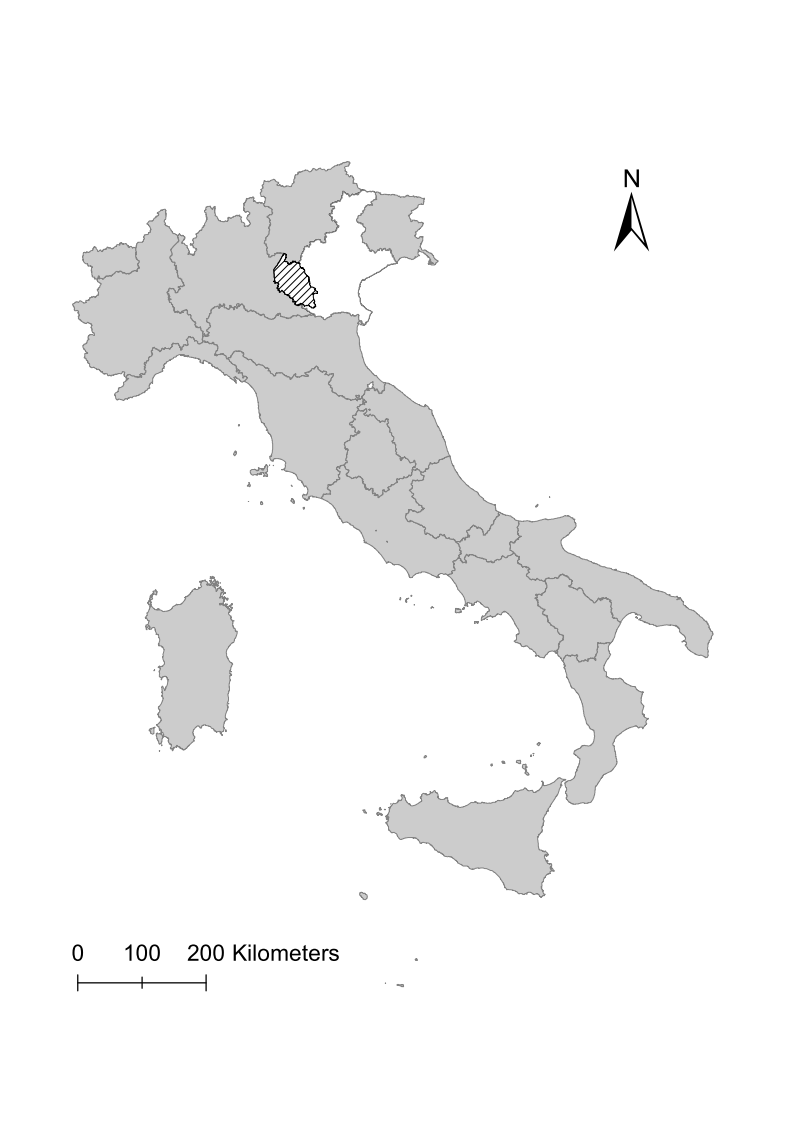


* the image was produced using Arc Map 10.1 software (ESRI, Redlands, California)

**Table S1**. Review of reference guidelines on the management of diabetes available in 2006. Guidelines reporting performance indicators (1-3) were rated by three independent reviewers, who assigned a score from 0 (low) to 3 (high) to each of three items: I. multidisciplinary panel; II. evidence search strategy reported; III. grading system reported [16].

| **N** | **Title** | **Provider** | **Year** | **Indicators**  **availability** | **Score** | | | | **Mean score** |
| --- | --- | --- | --- | --- | --- | --- | --- | --- | --- |
|  |  |  |  |  | Item | Reviewer | | |  |
|  |  |  |  |  |  | 1 | 2 | 3 |  |
| 1 | Clinical Guidelines for Type 2 Diabetes | National Institute for Clinical Excellence | 2005 | Yes | I | 2 | 2 | 2 | 2.4 |
|  |  |  |  |  | II | 2 | 2 | 3 |  |
|  |  |  |  |  | III | 3 | 3 | 3 |  |
| 2 | Diabetes Guideline | Prodigy | 2005 | Yes | I | 0 | 0 | 0 | 0.7 |
|  |  |  |  |  | II | 1 | 1 | 1 |  |
|  |  |  |  |  | III | 0 | 0 | 3 |  |
| 3 | Management of Type 2 Diabetes | New Zealand Guidelines Group | 2003 | Yes | I | 2 | 2 | 2 | 2.0 |
|  |  |  |  |  | II | 1 | 1 | 1 |  |
|  |  |  |  |  | III | 3 | 3 | 3 |  |
| 4 | Wisconsin Essential Diabetes Mellitus Care Guideline | Wisconsin Diabetes Advisory Group | 2004 | No |  | | | |  |
| 5 | 2006 Clinical Practice Recommendations | Diabetes Care | 2006 | No |  | | | |  |
| 6 | Massachusetts Guidelines for Adult Diabetes Care | Produced by the Massachusetts Health Promotion Clearinghouse | 2005 | No |  | | | |  |
| 7 | Basic guidelines for diabetes care | Diabetes Coalition of California | 2005 | No |  | | | |  |
| 8 | Management of Type 2 Diabetes | Institute for Clinical Systems Improvement | 2005 | No |  | | | |  |
| 9 | Medical Guidelines for the Management of Diabetes Mellitus | American Association of Clinical Endocrinologists | 2002 | No |  | | | |  |
| 10 | Management of Type 2 Diabetes Mellitus | University of Michigan | 2006 | No |  | | | |  |
| 11 | Management of Diabetes Mellitus | Michigan Quality Improvement Consortium Guidelines | 2004 | No |  | | | |  |
| 12 | Recommendations for management of diabetes in Vermont | Vermont Department of Health | 2004 | No |  | | | |  |
| 13 | Guidelines for the Management of Adult Diabetes in Primary Care | Kaiser Permanente | 2005 | No |  | | | |  |
| 14 | Clinical practice guideline for the management of diabetes mellitus in primary care | VA/DoD | 2003 | No |  | | | |  |
| 15 | Diabetes Care | British Columbia Ministry of Health and Medical Association | 2005 | No |  | | | |  |
| 16 | Diabetes Mellitus | British Medical Association | 2004 | No |  | | | |  |
| 17 | Clinical Practice Guidelines for the Prevention and Management of Diabetes in Canada | Canadian Diabetes Association | 2003 | No |  | | | |  |
| 18 | Diabetes Commissioning Toolkit | National Health System | 2006 | No |  | | | |  |
| 19 | Diabetes | Department of Health | 2006 | No |  | | | |  |
| 20 | Practice guidance on the care of people with diabetes | Royal Pharmaceutical Society | 2004 | No |  | | | |  |
| 21 | Diabetes Mellitus | Ministry of Health Singapore | 2006 | No |  | | | |  |

**Table S2**: number (percentage) of patients with diabetes stratified by quality of care score (Q-score) in 2006 and 2009.

| **Q-score** | **2006**  (N=14480) | **2009**  (N=14162) |
| --- | --- | --- |
| 0-10 | 79 (1%) | 80 (1%) |
| 15-20 | 12162 (84%) | 10692 (75%) |
| 25-40 | 2239 (15%) | 3390 (24%) |
